# Characteristics of fixation patterns and their relationship with visual function of patients with idiopathic macular holes after vitrectomy

**DOI:** 10.1038/s41598-021-87286-9

**Published:** 2021-04-07

**Authors:** Yuyan Liu, Ying Wang, Yi Dong, Dongqing Liang, Shiyong Xie, Bo Xiao, Yanhua Chu, Quanhong Han

**Affiliations:** 1 Tianjin Eye Hospital, Tianjin Key Lab of Ophthalmology and Visual Science, Tianjin Eye Institute, Nankai University affiliated Eye Hospital, Clinical College of Ophthalmology, Tianjin Medical University, Gansu Road 4, Heping District, Tianjin, 300020 China; 2 Tianjin Medical University, Tianjin, China

**Keywords:** Diseases, Signs and symptoms

## Abstract

To analyze the relationships between the fixation location and the visual function of idiopathic macular hole (IMH) patients with macular integrity assessment (MAIA) examination preoperatively and 3 months postoperatively. This was a retrospective case analysis. Forty-three eyes of 43 patients diagnosed with IMH were included in this study. The best corrected visual acuity (BCVA) assessments, optical coherence tomography (OCT) and MAIA examinations were performed before surgery and 1 week, 1 month and 3 months after surgery. The relationships between MAIA parameters and visual acuity were assessed by correlation analysis. Grouping by fixation location with the foveola (2°) as the centre, the locations could be divided into five groups, including foveolar, temporal, nasal, inferior and superior fixation. The mean macular sensitivity (MMS) of the macular area was correlated with the BCVA in the IMH patients before and 3 months after surgery (before surgery *P* = 0.00, after surgery *P* = 0.00). The MMS could be used as a good indicator for evaluating visual function in IMH patients. There was a significant difference in fixation location before and after the operation (*P* = 0.01). The preoperative fixation location of IMH patients was mainly in the superior area, while postoperatively moved to the foveola and nasal areas. Paying attention to the changes of fixation locations in IMH patients may provide new clues for further improving postoperative visual function.

## Introduction

Idiopathic macular hole (IMH) is a common retinal disease that mostly affects elderly people^1^. The perpendicular or tangential traction generated by the vitreous and the inner limiting membrane to the retinal surface can be the causes of IMH formation^2,3^. In 1991, Kelly and Wendel first reported that vitrectomy was effective in the treatment of IMH^4^. Pars plana vitrectomy (PPV) is the most effective treatment of full-thickness IHM, and most untreated patients will progress in size and grade and lead to increasing central visual loss^5^. Recently, PPV with internal limiting membrane (ILM) peeling or the inverted ILM flap technique has been used to achieve successful hole closure in 90–100% of IMH patients and visual improvement in over 85% of patients^6–8^. However, postoperative visual function is unpredictable after surgery despite anatomic closure^9^.

Although difficult, attempts have been made to explain the discrepancy between the anatomic and functional results^9^ by examining the abnormalities found in the macular morphology as revealed by optical coherence tomography (OCT)^10^, but some visual outcomes still cannot be predicted by morphologic characteristics.

In addition to differences in surgical techniques, the microstructures of the macular area can affect postoperative visual acuity. Importantly, the fixation locations of IMH patients also change after the operation^11^. With the improvement in microperimetry technology, the study of IMH fixation locations after surgical treatment has become more accurate. Previous studies have shown that after surgery, most IMH patients convert to foveal fixation^9,11^. Additionally, three articles indicated that in view of the limitations of the examination technology at that time, there may be deviations in the detection of fixation locations. Using the current macular integrity assessment (MAIA) examination method, we can more accurately describe the fixation locations, and determine the characteristic of the changes of the fixation pattern before and after surgery. This is of great clinical significance, because the fixation location is closely related to visual acuity, and MAIA biofeedback training has been shown to improve visual acuity in patients with insufficient recovery of their best corrected visual acuity (BCVA) after successful IMH surgery^12^. The main purpose of this study was to observe the patterns of change in the fixation location in IMH patients before and after surgery and to assess the relationship between the fixation pattern and visual function.

## Results

### General characteristics

The patient characteristics are shown in Table 1. IMH occurred in the right eye in 18 patients and in the left eye in 25 patients. The mean age of the patients was 63.60 ± 5.82 years (range, 48–76 years). The mean duration of symptoms was 5.24 ± 7.90 months. The IMH was classified as stage 2 in 8 eyes, stage 3 in 6 eyes, and stage 4 in 29 eyes according to the Gass classification^13^. The macular holes were closed for all of the 43 patients.

**Table 1:** Baseline demographic and clinical characteristics of the 43 participants.

Parameters	Values
Age(years)(mean ± SD; range)	63.60 ± 5.82; 48–76
**Gender, n (%)**	
Male	8(18.60)
Female	35(81.40)
**Eye, n (%)**	
Right	18(41.86)
Left	25(58.14)
Axial length (mm) (mean ± SD; range)	23.53 ± 1.08; 21.60–25.55
Refractive status (D) (mean ± SD; range)	− 0.56 ± 2.22; − 5.75- + 1.75
Preoperative logMAR BCVA (mean ± SD; range)	1.10 ± 0.51; 0.30–3.0
Symptom duration (months) (mean ± SD; range)	5.24 ± 7.90; 0.20–36
**Preoperative stage, n(%)**	
2	8 (18.60)
3	6 (13.95)
4	29 (67.44)
Preoperative MLD of the hole (μm) (mean ± SD; range)	465.36 ± 186.51; 190.00–883.00
Preoperative BD of the hole (μm) (mean ± SD; range)	904.00 ± 301.86; 482.00–1540.00
Preoperative H of the hole (μm) (mean ± SD; range)	422.52 ± 83.57; 264.00–600.00
Preoperative CCT (μm) (mean ± SD; range)	214.00 ± 58.31; 99.0–352.00
Postoperative logMAR BCVA (mean ± SD; range)	0.50 ± 0.34; 0–1.30

*BD* base linear diameter, *BCVA* best-corrected visual acuity, *CCT* central choroid thickness, *H* height (H) of the macular hole, *logMAR* logarithm of the minimal angle of the resolution scale, *MLD* minimum linear diameter, *SD* standard deviation.

### Correlations of MAIA parameters with BCVA before and after surgery

**Figure 1:**
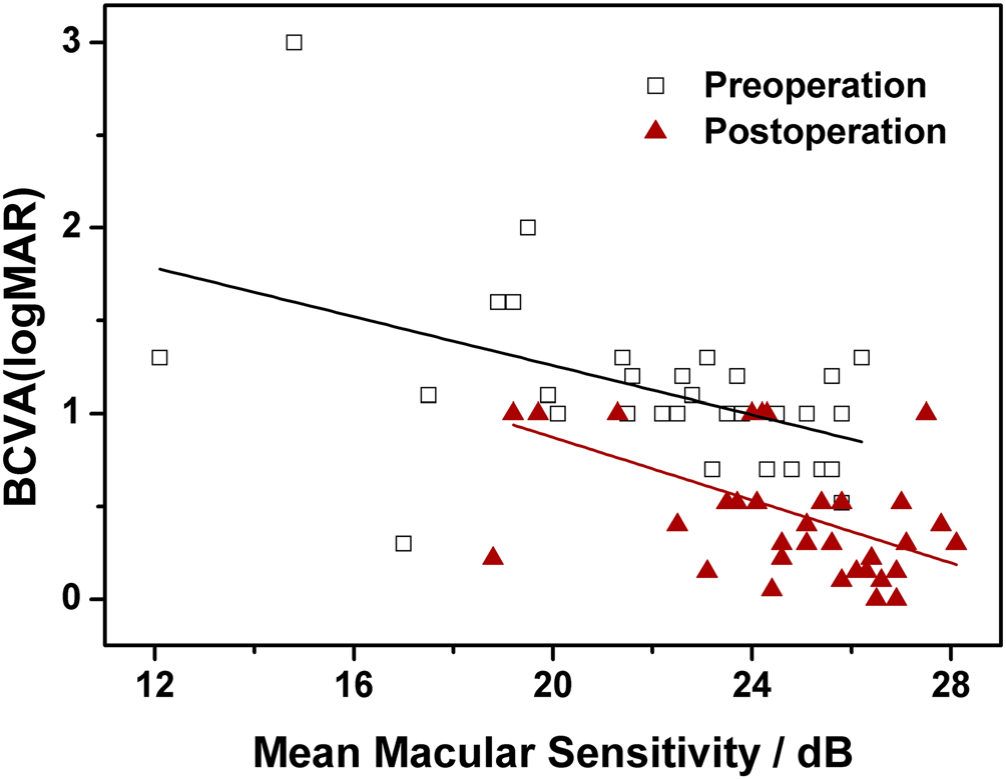
Association between the best-corrected visual acuity (BCVA) with mean macular sensitivity (MMS) before and after surgery. The “□” symbols represent preoperative visual acuity and MMS data, and the red “▲” symbols show the postoperative visual acuity and MMS data. The solid lines represent the linear regression curves for the data obtained before and after surgery.

Spearman analysis was conducted between preoperative visual acuity and MAIA parameters, and the results are shown in Table 2. Before surgery P1 and MMS (Fig. 1) were significantly correlated with preoperative BCVA (*P* = 0.03, 8.44E−3, respectively), but P2, the 63% bivariate contour ellipse area (BCEA) and 95% BCEA before surgery were not (*P* = 0.57, *P* = 0.17, *P* = 0.20, respectively).

**Table 2:** Results of Spearman analyse between MAIA parameters and BCVA before and after surgery.

Parameters	Mean ± SD (Pre-O)	*P* value (Pre-O)	Mean ± SD (Post-O)	*P* value (Post-O)
P1 (%)	69.73 ± 17.83	*P* = 0.03	89.15 ± 11.76	*P* = 0.08
P2 (%)	93.62 ± 6.57	*P* = 0.57	98.00 ± 2.49	*P* = 0.90
MMS (dB)	22.05 ± 3.22	*P* = 2.97E−3	25.55 ± 2.19	*P* = 8.44E−3
63%BCEA	3.37 ± 2.16	*P* = 0.17	1.32 ± 1.17	*P* = 0.37
95%BCEA	10.1 ± 6.45	*P* = 0.20	3.94 ± 3.50	*P* = 0.37

P1 and P2 represent the number of fixating points which was in circles of 1° and 2°radius, respectively.

*MMS* mean macular sensitivity, *BCEA* bivariate contour ellipse area, *Pre-O* preoperation, *Post-O* postoperation, *SD* standard deviation.

Table 2 also shows the results of Spearman analyse between postoperative visual acuity and MAIA parameters 3 months after surgery; similar results were obtained, that is, a significant correlation was shown between postoperative MMS (Fig. 1) and postoperative BCVA (*P* = 8.44E−3), while the other MAIA parameters were not**.**

MMS and the minimum linear diameter (MLD) were considered predictive factors for postoperative visual acuity in IMH patients in previous studies^14^. In this study, the correlation between preoperative MMS and MLD was statistically significant (*P* = 2.96E−4, Spearman analysis), and both preoperative MMS and MLD were correlated with BCVA 3 months postoperatively (MMS: *P* = 0.02; MLD: *P* = 1.16E−4, respectively, Spearman analysis).

### The relationship between fixation location and visual function

**Figure 2:**
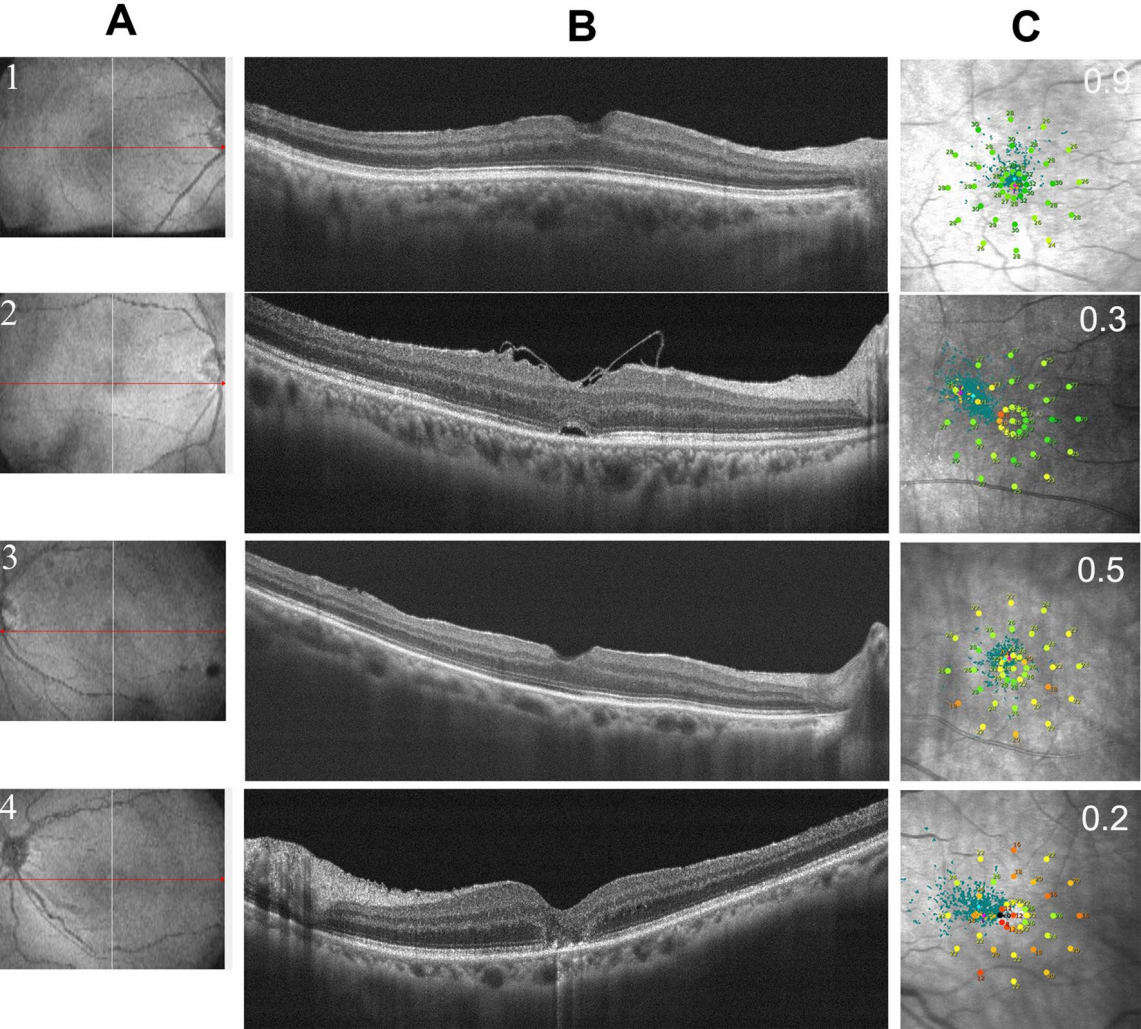
Results of OCT and MAIA examinations in patients with different fixation locations 3 months after surgery. Columns (**A**) and (**B**) show the location of the OCT scan and the horizontal B scan, and column (**C**) shows the MAIA results of the corresponding patients. The first row shows the OCT and MAIA results of patients with foveolar fixation locations, and rows 2, 3 and 4 show the results of patients with temporal, superior, and nasal fixation locations, respectively.

Because of the damage to central visual function in IMH patients, the fixation location before surgery was the parafoveola region. Although the macular hole was closed for most patients after the operation, some patients were still unable to shift to foveolar fixation after the operation (Fig. 2).

**Figure 3:**
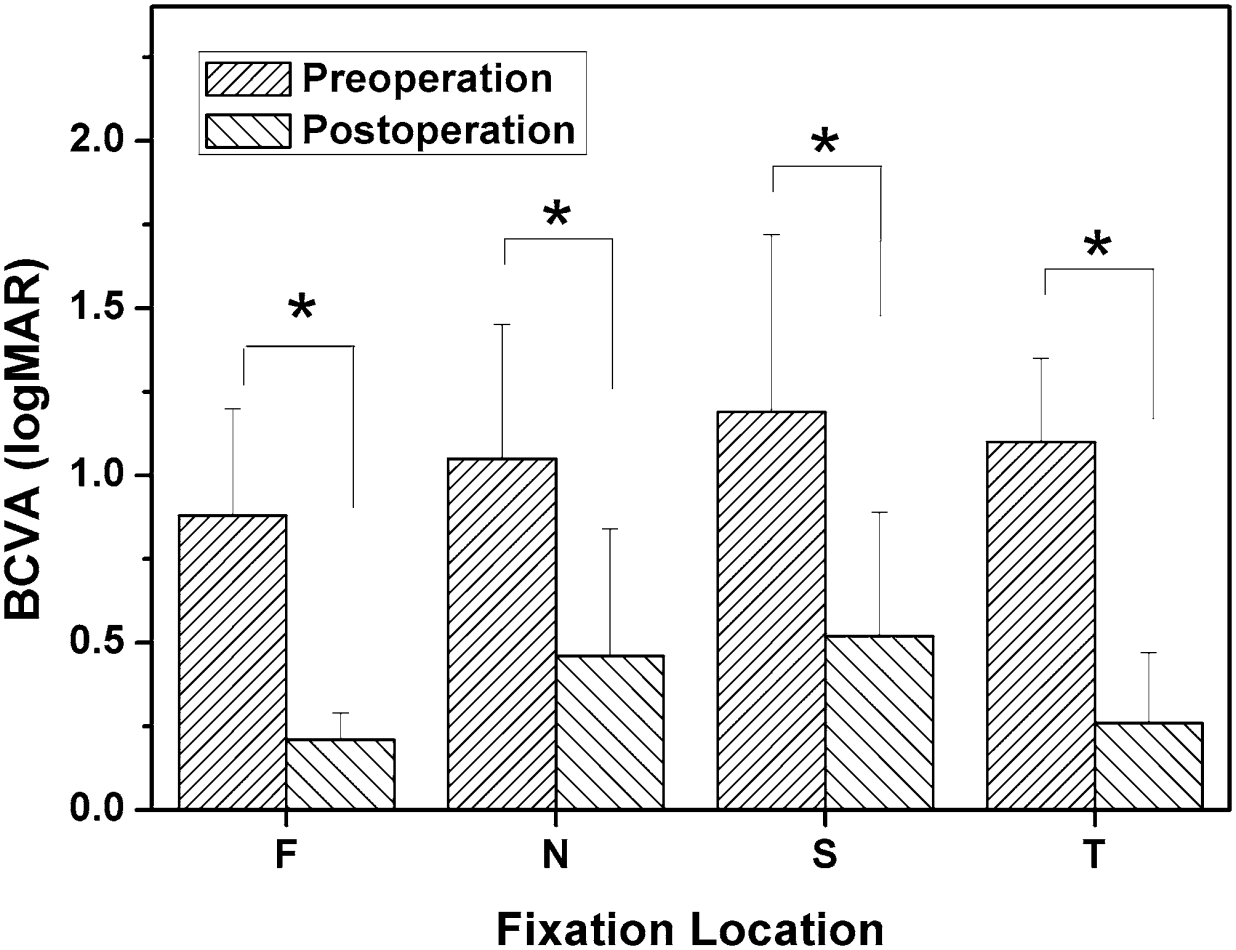
Comparison of preoperatively and postoperatively best corrected visual acuity (BCVA) between different groups according to the preoperative fixation location (F: foveola fixation group; N: nasal fixation group; S: superior fixation group; T: temporal fixation group).

The BCVA before and after surgery in different fixation locations grouped by preoperatively fixation location is shown in Fig. 3 (F: pre-op: 0.88 ± 0.32, post-op: 0.21 ± 0.08; N: pre-op: 1.05 ± 0.40, post-op: 0.46 ± 0.38; S: pre-op: 1.19 ± 0.53, post-op: 0.52 ± 0.37; T: pre-op: 1.10 ± 0.25, post-op: 0.26 ± 0.21). According to the results of the Mann–Whitney U test, there were significant improvements in BCVA for each fixation group after surgery (F: *Z* = − 2.32, *P* = 0.02; N: *Z* = − 2.65, *P* = 0.01; S: *Z* = − 4.22, *P* = 2.43E−5; T: *Z* = − 2.89, *P* = 0.04, respectively). The results showed a trend towards better visual acuity in patients with foveolar fixation, but there was no statistically significant difference in BCVA grouped by fixation location before and after surgery (before surgery: *χ*^2^ = 1.43, *P* = 0.70; after surgery: *χ*^*2*^ = 4.10, *P* = 0.25; Kruskal–Wallis analysis).

The primary preoperative fixation locations of the IMH patients were the superior and temporal areas, while the postoperative fixation locations were on the foveolar and nasal areas (Table 3). There were no patients whose fixation was located in the inferior area before and after the operation. The differences in fixation location distributions before and after surgery were statistically significant (*χ*^*2*^ = 11.94, *P* = 0.01, Table 3).

**Table 3:** Distribution of fixation locations before and after surgery.

	Post-Nasal	Post-Superior	Post-Temporal	Post-Foveola	Pre-Total
Pre-Nasal	8	0	0	1	9
Pre-Superior	6	10	1	6	23
Pre-Temporal	0	1	1	4	6
Pre- Foveola	0	1	0	4	5
Post-Total	14	12	2	15	43

## Discussion

When the macular hole of IMH patients is successfully closed after surgery, improvement in visual function is an important concern for both vitreoretinal specialists and patients. Both the microstructures of the macular area and the changes in fixation locations can affect postoperative visual acuity. Microperimetry is an essential technique for assessing the morphological and functional losses in macular pathologies^15,16^, and can provide retinal sensitivity and fixation stability for retinal function assessment^14^. Witha scanning laser ophthalmoscope (SLO), we can precisely measure retinal sensitivity and determine which part of the retina the patient is using for fixation^11^. MAIA has evolved into a robust tool for evaluating retinal function in recent years^14,17^. It can be used to map the location of scotomas and the results can be accurately plotted by correcting for eye movements. This could provide detailed and precise information for retinal function changes before and after surgery.

This study showed that MMS is negatively correlated with visual acuity in the 10 degree MAIA test mode in IMH patients, and that it can be used as one of the main indicators of visual function before and after IMH surgery, which is consistent with previous reports^14,15^. Studies on MAIA in normal subjects show that the MMS and P1 measurements are relatively stable, while the other MAIA parameters can vary greatly^18^. Tarita-Nistor et al. reported that a change in fixation stability was a strong predictor of visual outcome after successful closure of the macular hole^9^. Therefore, the description of the MAIA parameters is of great significance in the evaluation of visual function in IMH patients.

Our study showed that the BCVA was not significantly different between IMH patients with different fixation locations before and after surgery, possibly due to the small number of patients in the analysis. In previous studies, IMH patients have demonstrated paracentral fixation, and after surgery some showed central fixation^11,14,19–21^. In our study, among 43 patients, central fovea fixation was found in 5 patients preoperatively and 15 postoperatively. A previous study showed that all patients showed paracentral fixation preoperatively, and 27 patients (27/44) shifted to foveolar fixation 4 months after surgery^14^. The inconsistency in the ratio of foveolar fixation after surgery between previous results and this report may be due to the different follow-up times.

Further analysis of the results of this study showed that the preoperative fixation location was mainly in the superior area in IMH patient, and moved postoperatively to the nasal side and central fovea^9^. Theoretically, the ideal result after successful macular hole closure would be recovery of macular function and movement of the preferred retinal locus (PRL) moves back to its former location; however, this may not always be the case^22^. Guez et al.^23^, who visually inspected the PRL shift using an SLO, reported that the PRL became central after surgery in a high proportion of cases; however they acknowledged that: due to measurement and experimenter errors, the new location might actually have been paracentral^9^. With the development of microperimetry technology, these paracentral locations can be gradually recognized.

This study found that the fixation location shifted to the foveolar and nasal side of the macula after surgery. Recent studies have shown that the macula fovea in IMH patients shifts between 100 and 300 µm to the nasal side and slightly downward after ILM removal^22,24,25^. Foveal displacement following MH surgery has also been reported^12,26^. Ishida et al. showed that the ratio of retinal displacement in the temporal field was significantly correlated with the basal diameter of the MH^27^. We concluded that fixation location displacement might be one of the reasons for poor visual recovery after successful MH surgery. Helping patients to fixate at the point with a better MS after MH closure might improve their visual function^12^.

The study has some limitations. First, this was a retrospective study with a limited number of participants, which might be the reason why no significant differences were found in visual function between the fixation patterns. Second, the same surgeon operated on all patients, so the results may have some limitations. Third, this article focuses on fixation location, while other preoperative and postoperative clinical parameters that could be related to the outcomes were not fully discussed. Finally, the duration of the follow-up period was only three months.

In conclusion, MMS is closely related to visual acuity and may be a reliable indicator of visual function. The preoperative fixation location of IMH patients was mainly in the superior area, while the postoperative fixation location moved to the foveola and nasal sides. The patients in the foveolar fixation group showed better visual acuity than those in the other groups, but the difference in BCVA grouped by fixation location was not statistically significant. Further prospective multicentre studies including a larger patient sample and a longer follow-up could provide more reliable conclusions.

## Methods

### Participants

The study was a retrospective, observational case series. The described research adhered to the tenets of the Declaration of Helsinki. The research was conducted in Tianjin Eye Hospital and approved by the Institutional Review Board/Ethics Committee of Tianjin Eye Hospital (TJYKYYLL-201926), which waived the need for written informed consent from the subjects due to the retrospective nature of the study.

Patients with full-thickness macular holes (stages 2, 3, 4) on OCT examination who underwent 25-gauge (25G) pars plana vitrectomy (PPV) with the inverted ILM flap technique in Tianjin Eye Hospital between January 2015 and August 2017 were included. The stage of the IMH was classified according to the Gass classification^13^. Patients with secondary or traumatic macular holes, myopic eyes with a refractive error of less than − 6.00 dioptres, age-related macular degeneration, proliferative diabetic retinopathy, and solar retinopathy were excluded. Fifty-five participants were included in this study.

There were four patients who could not successfully complete the MAIA examination due to poor visual acuity. Six patients were excluded because of the presence of retinal diseases. Two patients did not return for follow-up for at least 3 months. Therefore the results of 43 eyes from 43 patients (8 men and 35 women) were used in the statistical analysis.

A detailed eye examination including slit-lamp examination (BQ 900, HAAG-STREOT AG, Swiss), fundus examination by indirect binocular ophthalmoscopy (SL4 4AA, Keeler, USA), and fovea microstructure examinationed by spectral domain OCT (SD-OCT) (RTVue XR 100-2, Optovue, USA) was performed. Preoperative data included age, sex, symptom duration, right or left eye, BCVA (international visual chart), axial length (AL), refractive status, MLD, height (H), base linear diameter (BD) of the macular hole, and central choroid thickness (CCT)^28^. BCVA assessments, OCT and MAIA examinations were performed 1 week, 1 month and 3 months after surgery.

### MAIA examinations

Each participant was properly instructed on how to perform the technique before the first examination^18^. All measurements were performed in a dark room after 10 min of dark adaptation without any pupil dilation^16^. All patients were comfortably positioned properly on the device with the chin on the chin pad and the finger ready to press a response trigger. The MAIA (Centervue, Padova, Italy) instruments used a 37-stimulus grid overlying the central 10° of the visual field. The standardized stimulus grid consisted of a single central foveal response and three concentric rings of retinal loci at 1°, 3° and 5° from the centre point. The MAIA measurement was carried out by a 4-2-1 staircase strategy with the Goldmann III stimulus size. The background luminance was 4 asb. The maximum luminance of the MAIA device was 10,000 asb, the minimum luminance was 0.25 asb, the luminance duration was 200 ms and the stimulus dynamic range was between 0 and 36 dB^16^.

As described in a previous article, P1 and P2 represent the total number of fixating points within circles of 1° and 2° respectively. Another parameter used to characterize the fixation pattern is the BCEA which represents the area in square degrees of the ellipse that includes either 63% or 95% of the fixation positions^18^. The MMS is the mean sensitivity of the central 37 loci (enclosed by a circle with a 10°diameter)^29,30^.

**Figure 4:**
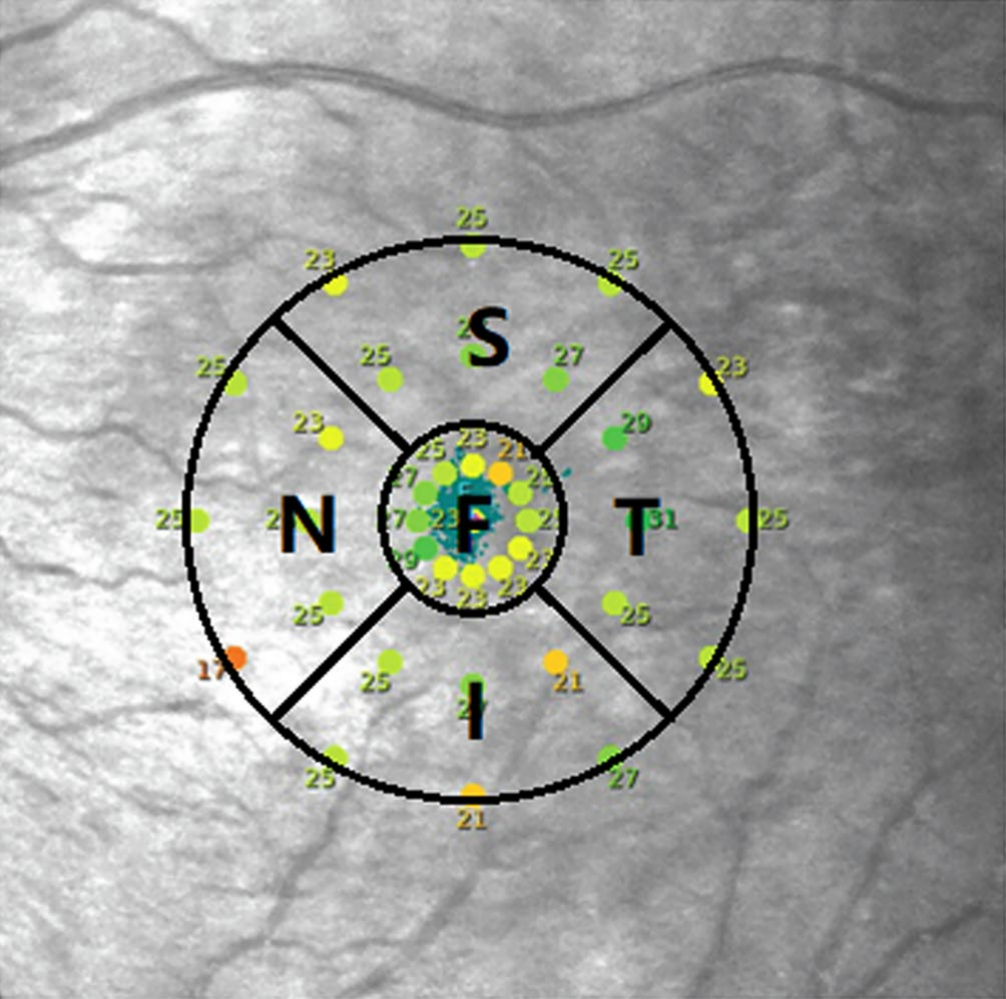
Division diagram of the fixation location based on microperimetry examination results. The inner circle and outer circle represent the 2° and 10° fixation ranges of the macular area respectively. The lines between the two circles are ± 45° with the horizontal line. Fixation locations beyond the foveola were divided into superior, inferior, nasal and temporal fixation locations.

The instrument records the fixation location using an auto eye-tracking system that identifies eye positions relative to an anatomic landmark. The automatic funds eye-tracking system enables the accurate projection of stimuli to ensure the same points on the retina during the follow-ups^9^. We set the macular hole as the fixation centre before surgery during the MAIA examination, and focal fixation was mainly located in the 2° range, which corresponds to the position of the foveola^31^. According to fixation location, patients were divided into superior (S), inferior (I), nasal (N), temporal (T) and foveolar (F) fixation locations (Fig. 4).

### Surgical procedure

All surgeries included in the study were performed by the same vitreoretinal specialist (H. QH.). A standard sutureless (25 G) 3-port PPV was performed for all patients, including the induction of posterior vitreous detachment when required. An intravitreal injection of indocyanine green (5 mg/mL) was performed to better visualize the ILM. The ILM was peeled off in a circular fashion for approximately 2 disc diameters around the macular hole, and the remaining ILM around the macular hole was trimmed short and massaged gently over the MH until the ILM became inverted^7,32^. Phacoemulsification with implantation of the intraocular lens and circular dissection of the posterior capsule was performed simultaneously for patients with cataracts or those older than 50 years. Sterile air was used to tamponade the vitreous cavity, and patients were instructed to maintain a prone position while awake for at least 4 days postoperatively.

### Statistical analysis

For statistical analysis, the BCVA was converted to logarithm of the minimal angle of the resolution scale^33^ (logMAR) units. The parameters are presented as the mean ± standard deviation (SD). Comparisons of data between different groups were analysed with the Kruskal–Wallis test. Spearman analysis was performed to investigate the correlation between BCVA and MAIA parameters. Statistical analysis was performed using SPSS statistics software, version 19.0 (IBM, Armonk, New York, USA). Differences were considered significant at *P* < 0.05.
